# Recent Meeting of the American Medical Association

**Published:** 1871-05

**Authors:** 


### Editorial.

### Recent Meeting of the American Medical Association.

We devote as much space as possible to the re-publication of a part of the proceedings of the meeting of the American Medical Association in San Francisco. That there were very valuable medical papers presented to the various sections, there can be no doubt, and that the transactions of the Association will be the medium of communicating to the profession the results of recen- and original investigation and study; while it is not so certain that the legislative action of this body was really of any service or credit to either the profession or public. The resolutions on the subject of distinct chair s of Hygiene, or other professional chairs in our Medical Colleges, can hardly be regarded as likely to be of any avail; while the discussion of the question of admitting women to all the rights and privileges of the profession, appears quite crude and unconsidered, in its detail and aggregate, taking from, rather than adding to the respect which is generally entertained for the profession. It will be time to consider this question when any considerable number of women comply with the usual terms of graduation in this country, and actually become well qualified, intelligent and trustworthy physicians. The general fact that most of the female practitioners of medicine, are, at present, little more than imperfectly educated nurses, and are likely to generally be found of this class for a long time to come, is not to be placed as a bar to any woman who actually does study, under proper instruction, the requisite time, and finally graduates in medicine with the proper qualification. Whenever *such* female physicians can be met in consultation, sex cannot, with any show of fairness be urged as ground of denial of the civilities and courtesies which all the cultivat tors of medical science naturally, instinctlvely and gladly extend to all other faithful, intelligent and earnest workers in the same great field of human knowledge. Educated minds have an "affinity" which race, and nation, and color, and *sex* cannot change. Show us an intelligent, experienced, honest and earnest female physician, who faithfully pursues the calling, with modesty and courtesy, and if invited to do so, we propose to meet her and not be greatly outdone in professional politeness. But, alas! too many never pass through the door into the true "sheep fold," but climb up some other way. These are medical thieves and robbers of which all should beware.

These local quarrels, which last year had origin in Washington, in the negro hatred; and this year in Philadelphia in the question of the admission of female physicians to consultations, are neither profitable or creditable to the American Medical Association, and if such questions are allowed year after year to engage the attention, and the discussions are published, in full, in the daily papers of the country, the intelligent public, as well as the great body of fair-minded physicians, will come at length to look upon its meetings as only affording opportunity for expression of personal or local prejudice or jealousy or passion, and of no value to truth or science or professional use fulness, or any other praiseworthy object. The meeting in San Francisco appears to have been greatly enjoyed and a larger delegation from tne Atlantic States was present than could have been expected. The hospitality of the profession of California was characteristic of the earnest men who have so recently manifested their resolute purposes by settlement in this new world. The ride to the Pacific, the incidents of so long a journey, the warm reception, together with the many objects of attraction, all, we understand, made the meeting one of the most pleasant ever held by the Association.

